# Quantitative determination of trace principal components with high specific activity in menotropins

**DOI:** 10.3389/fbioe.2026.1783311

**Published:** 2026-05-04

**Authors:** Yue Sun, Heyang Li, Xinyue Hu, Shuqing Luo, Xiaoming Zhang, Lyuyin Wang, Yi Li, Ping Lyu, Jing Li

**Affiliations:** 1 National Institutes for Food and Drug Control, State Key Laboratory of Drug Regulatory Science, NMPA Key Laboratory for Quality Research and Evaluation of Chemical Drugs, Beijing, China; 2 Chemical Drug Analysis Laboratory, Ningbo Institute for Drug Control, Ningbo, China

**Keywords:** biological potency, menotropins, purity assessment, quantification, α-subunit, β-subunit

## Abstract

**Introduction:**

Menotropins is a mixture of gonadotropin glycoproteins extracted from the urine of postmenopausal women. Its composition is complex, with active components including follicle-stimulating hormone (FSH), luteinizing hormone (LH), and human chorionic gonadotropin (hCG), in addition to many unknown components. The current standards for menotropins rely exclusively on in vivo animal models for the bioactivity control of FSH and LH. However, these methods are complex and highly variable. Moreover, they fail to accurately quantify the high specific bioactivities of FSH and LH, which in turn impairs the batch-to-batch consistency of the final product. To date, there is no appropriate method for accurately quantifying these trace principal components with high specific activities.

**Methods:**

A reversed-phase high-performance liquid chromatography (RP-HPLC) method was established in the present study to quantify the α-subunits content common to FSH, LH, and hCG, as well as the FSH content, while simultaneously enabling purity assessment in menotropins and their formulations. The α-subunit peaks of FSH, LH, and hCG and the β-subunit peak of FSH were selected as the target peaks, with recombinant human FSH (rhFSH) employed as the external standard for quantification. Purity was calculated as the percentage of the sum of the α- and β-subunit peak areas using the area normalization method in chromatographic analysis. The validation procedure strictly followed ICH Q2(R1) guidelines and the requirements of the Chinese Pharmacopoeia, with evaluations of specificity, precision, linearity,accuracy, robustness, stability, limit of detection (LOD), and limit of quantification (LOQ). In addition to developing this HPLC method, this study conducted large-scale real-world sample analysis and head-to-head comparisons with the animal assay.

**Results:**

Methodological validation demonstrated that the developed RP-HPLC assay possessed excellent linearity, with correlation coefficients (R^2^) exceeding 0.998 for both subunits. The accuracy was confirmed by recovery rates ranging from 90.0% to 110.0%, with relative standard deviations (RSD) consistently below 2.0%. The limit of detection (LOD) and limit of quantification (LOQ) were determined to be 3 ng and 9 ng, respectively, providing the requisite sensitivity for the detection of these trace bioactive components within complex matrices. The analysis of 69 commercial batches of menotropins, sampled across manufacturing, distribution, and clinical use stages, revealed significant insights into product consistency. While standard in vivo animal bioassays exhibited substantial inter-assay variability and lacked the sensitivity to detect subtle batch-to-batch fluctuations, the HPLC method provided precise and reproducible quantification of the α-subunits (common to FSH, LH, and hCG) and the FSH.

**Discussion:**

As a result of its operational simplicity, accuracy, and high sensitivity, this HPLC method enables the simultaneous quantification of the total α-subunits and FSH content in menotropins, as well as assessment of their purity. It successfully addresses the analytical challenges posed by the complex matrix, abundant non-target proteins, and extremely low content of active ingredients in conventional menotropins products. It thereby provides a robust analytical tool for enhancing product quality control and safety assurance.

## 1 Introduction

Human menopausal gonadotropin (hMG), also known as menotropins, is a mixture of gonadotropin glycoproteins extracted and purified from the urine of postmenopausal women. Menotropins exhibits the biological activity of follicle-stimulating hormone (FSH) and luteinizing hormone (LH), with the ratio of LH to FSH activity approximately 1:1 (Practice Committee of American Society for Reproductive MedicineBirminghamAlabama, 2008). When necessary, hCG obtained from the urine of pregnant women may be added to maintain this ratio. Clinically, it is used to treat primary or secondary amenorrhea, oligomenorrhea, and infertility due to insufficient gonadotropin secretion. Controlled ovarian stimulation (COS) is used in assisted reproductive technologies to induce multiple follicular development.

In 1950, Lunenfeld (2012) successfully extracted menotropins from the urine of postmenopausal women, using kaolin for adsorption and acetone for protein precipitation. Merck Serono registered the injectable form of menotropins, Pergonal®, in Israel in 1963 and in Italy in 1965. A similar formulation, Humegon®, was launched by Organon in the Netherlands in 1965. Currently, manufacturers in China with marketing approval for injectable menotropins include Yantai Dongcheng Northern Pharmaceutical Co., Ltd., Livzon Pharmaceutical Group Inc. (Livzon Pharmaceutical Factory), Ma’anshan Fengyuan Pharmaceutical Co., Ltd., and Shanghai Shangyao First Biochemical Pharmaceutical Co., Ltd. At present, there are numerous market-competing FSH-containing pharmaceutical preparations manufactured via recombinant DNA technology playing a key role in treating infertile women with anovulatory cycles or achieving COS in patients undergoing *in vitro* fertilization (IVF). Nevertheless, menotropins is still widely used and occupies a significant market share due to its superior ovarian tolerance, favorable cost-effectiveness, and frequent application in COS requiring LH activity supplementation (Revelli et al., 2015; Pacchiarotti et al., 2016; Bergandi et al., 2020; Chen et al., 2021; Khair et al., 2023; Yetkinel et al., 2024).

The composition of menotropins is complex, with active components that include FSH, LH, and hCG (Wolfenson et al., 2005). Additionally, it contains many unknown components. The active components of menotropins, including FSH, LH and hCG are members of the glycoprotein hormone family. Members of this family consist of a common α-subunit and a hormone-specific β-subunit that are non-covalently bound to form heterodimers (Cahoreau et al., 2015; Ulloa-Aguirre et al., 2018). This structural similarity presents a significant challenge for the accurate quantification of the individual active components. The specification for menotropins and its injectable formulations including the 2025 edition of the *Pharmacopoeia of the People’s Republic of China*, the 36th edition of the *United States Pharmacopeia*, the 17th edition of the *Japanese Pharmacopoeia*, and the 2025 edition of the *British Pharmacopoeia* rely solely on *in vivo* animal methods for FSH and LH potency control, which are complex, and highly variable. According to the 2025 edition of the *Pharmacopoeia of the People’s Republic of China* (Part III), the potency of rhFSH is specified as 10,500–16,500 IU/mg (Chinese Pharmacopoeia Commission, 2025). The specific activity of recombinant human chorionic gonadotropin (rhCG) is approximately 26,000 IU/mg (Leão and Esteves, 2014). The specific activity of recombinant human luteinizing hormone (rhLH) is approximately 25,000 IU/mg. Small variations in the content of FSH, LH and hCG protein (in the ng range) can lead to significant differences in activity, potentially producing different pharmacological effects or side effects. Thus the accurate determination of the three bioactive components is particularly important. Menotropins for injection is available in two strengths: 75 IU/vial and 150 IU/vial, with FSH being the high specific activity trace main component. The specification employs only animal-based bioassays to control the potency of FSH and LH. The Steelman–Pohley method, which is based on ovarian weight increase in immature female rats, is a standard procedure for measuring the biological potency of FSH products. The coefficient of variation of each assay is typically between 10% and 20%, meaning that the actual biological activity observed in experiments may vary from 80 to 120 IU when 100 IU of FSH product is administered (Driebergen and Baer, 2003; Lispi et al., 2023). LH bioactivity assay was performed using the rat seminal vesicle weight-gain assay. The crude *in vivo* bioassay methodologies fail to differentiate high specific activity components, leading to inaccurate quantification and compromised batch-to-batch consistency of biopharmaceutical products. The accurate dosage of menotropins is closely linked to clinically significant outcomes such as follicular development and embryo quality (Revelli et al., 2015; Pacchiarotti et al., 2016; Wolfenson et al., 2005; Lunenfeld et al., 2019); the heterogeneous nature of its complex composition and batch-to-batch variations in high specific activity components may underlie adverse reactions.

Furthermore, the Basic Technical Requirements for Multicomponent Biochemical Drug Injection (Trial Version) issued by the National Medical Products Administration (NMPA) stipulates that due to the complexity of multicomponent biochemical drugs, appropriate analytical methods should be developed to ensure the relative uniformity of product quality. Lispi et al. (2006) and Muqaku et al. (2024) employed the Lowry method and a Pierce 660-nm protein assay kit to determine total protein content in highly purified human menopausal gonadotropin (HP-hMG). However, these methods only measure total protein content without specificity; as the active components account for a very low proportion of the total protein, measuring total protein is insufficient to reflect the true content of the active ingredients. Lispi et al. (2006) also used enzyme-linked immunosorbent assay (ELISA) to determine the content of LH and hCG in highly purified HP-hMG, however, it is dependent on the quality of antibodies, with high variability, poor reproducibility, and high requirements for operator skills.

The complex matrix of menotropins—featuring abundant non-target proteins and trace principal bioactive components with high specific activity—necessitates developing an HPLC assay for quantifying bioactive protein content. This method ensures batch-to-batch consistency, quality control, safety, and stability of menotropins and its formulations. The present study established an analytical method which can efficiently separate the β-subunits of FSH, LH, and hCG, while achieving co-elution of their identical α-subunits. This method enables simultaneous quantification of total α-subunits content common to the three gonadotropins (FSH, LH, hCG) and FSH content in menotropins, using recombinant human FSH (rhFSH) as an external standard to target both α- and β-subunit peaks. Given that oxidized α-subunits are major activity-lowering impurities in glycoprotein hormones requiring stringent control (Butt et al., 1961; Nevelli et al., 2023), purity was assessed via area normalization—despite co-elution of oxidized species with other proteins—by calculating the percentage ratio of summed α- and β-subunit peak areas, thus establishing a purity control strategy. A total of 69 batches of domestic menotropins for injection, covering three circulation stages (manufacturers, distributors, and end-users), were collected. The α-subunits content of FSH, LH, and hCG, along with FSH content, was quantified using the validated HPLC method. Results were systematically compared with FSH and LH potency values derived from standard animal bioassays, with purity simultaneously assessed.

## 2 Materials and methods

### 2.1 Solvents and reagents

The following reagents were used in this study: rhFSH stock solution (Merck Serono, batch no. FSE2327, 0.5 mg/mL); rhLH stock solution (Merck Serono, batch no. BLCA23023, 1 mg/mL); rhCG reference standard (National Institutes for Food and Drug Control, batch no. 410021-201901, 250 μg/ampoule, 6400 IU/ampoule); menotropins reference standard (National Institutes for Food and Drug Control, batch no. 150524-201805; FSH, 220 IU/ampoule; LH, 203 IU/ampoule); HP-hMG reference standard (departmental sample, batch no. 2010655; content, approx. 1 mg/ampoule); bovine serum albumin (BSA) (Sigma Company); physiological saline (Huaxia Shengsheng Pharmaceutical Co., Ltd., Beijing); acetonitrile and formic acid (Thermo Fisher Scientific (China) Co., Ltd.); trifluoroacetic acid (TFA, Sigma-Aldrich); potassium dihydrogen phosphate, phosphoric acid, ammonium bicarbonate, hydrogen peroxide and methionine (China Pharmaceutical Chemical Reagents Co., Ltd.); dithiothreitol (DTT) and iodoacetamide (Sigma Company); RapiGest SF denaturant (Waters Company); trypsin (Promega Company); deglycosylation kit (Biolabs Company); menotropins for injection (manufacturer 1, 29 batches; manufacturer 2, 16 batches; manufacturer 3, 9 batches; manufacturer 4, 15 batches); menotropins raw material (manufacturer 1, 2 batches; manufacturer 2, 1 batch; manufacturer 3, 1 batch).

### 2.2 Instruments

This experiment was conducted using a Shimadzu LC-20AD HPLC system (Shimadzu, Miyakonojō, Japan); Waters UPLC I-Class/synapt G2-S QTOF mass spectrometer (Milford, MA, United States) was used to analyze menotropins components.

### 2.3 HPLC methods

#### 2.3.1 Reference standard and test solution preparation

Briefly, 0.2 mL of rhFSH stock solution was accurately pipetted and diluted with a 0.1%.

TFA solution to a concentration of 0.05 mg/mL to obtain the reference standard solution. Menotropins reference standard solution was prepared by reconstituting one vial of menotropins reference standard with 0.5 mL of 0.1% TFA solution. Briefly, 10 μL of rhLH stock solution was diluted with a 0.1% TFA solution to a final volume of 75 μL and mixed well to obtain the rhLH solution. One vial of rhCG reference standard was reconstituted with 0.5 mL of a 0.1% TFA solution and mixed thoroughly. Then, 20 μL of the solution was diluted with a 0.1% TFA solution to a final volume of 75 μL and mixed thoroughly to obtain rhCG reference standard solution. One vial of HP-hMG reference standard was reconstituted with 1.0 mL of a 0.1% TFA solution and mixed thoroughly to obtain a stock reference solution. Then, 60 μL of the stock solution was added to 940 μL of a 0.1% TFA solution and mixed well to obtain the HP-hMG reference standard solution.

Menotropins was prepared by precisely weighing an appropriate amount of menotropins and dissolving it in a 0.1% TFA solution to a concentration of 18 mg/mL. Menotropins for injection was prepared by dissolving 1 vial of menotropins for injection in 0.5 mL of a 0.1% TFA solution to obtain a solution with a FSH concentration of 150 IU/mL.

Briefly, 0.05 mL of the rhFSH stock solution was added to 0.45 mL of water, followed by 10 μL of a 1.2% hydrogen peroxide solution. The solution was incubated at 37 °C for 40 min, and the reaction was terminated by adding 50 μL of a 25 mg/mL methionine aqueous solution, which was employed as the system suitability solution.

#### 2.3.2 HPLC method validation

To evaluate specificity, blank solvent (0.1% TFA solution), an excipient blank solution (prepared according to the formulation ratio of each manufacturer’s excipients), rhFSH solution, rhCG reference standard solution, rhLH solution, menotropins reference standard solution were injected for analysis.

To evaluate precision, menotropins for injection sample solution with a concentration of 0.01 mg/mL was injected consecutively 5 times, and the relative standard deviation (RSD) of the peak area of the α- and β-subunit were calculated.

To evaluate linearity range, a total of 200 μL of an rhFSH stock solution was diluted with a 0.1% TFA solution to a volume of 2 mL to obtain a linearity stock solution with a concentration of 0.05 mg/mL. Aliquots of 50, 100, 150, 200, 400, and 600 μL of the stock solution were separately diluted with a 0.1% TFA solution to a final volume of 1 mL, yielding linear sample solutions with concentrations of 0.0025, 0.005, 0.0075, 0.01, 0.02, and 0.03 mg/mL, respectively.

To evaluate recovery, accurately pipetted aliquots of 560, 800, and 1,040 μL of the linearity stock solution were separately diluted in a 0.1% TFA solution to a final volume of 4 mL, resulting in solutions with concentrations of 0.007, 0.01, and 0.013 mg/mL, respectively. Equal volumes of these solutions were then mixed with the menotropins for injection solution. Each concentration was prepared in triplicate to serve as spiked recovery solutions at concentrations of 70%, 100%, and 130%.

To evaluate robustness, the menotropins for injection solution was analyzed using three different chromatographic columns: Hichrom Vydac 214TP54 C4 column (4.6 mm × 250 mm, 5 μm; serial no. H78961), Hichrom Vydac 214TP54 C4 column (4.6 mm × 250 mm, 5 μm; serial no. H88219), and Waters Xbridge C4 column (4.6 mm × 250 mm, 5 μm; serial no. 01543919016119). The RSD of the α- and β-subunit contents were calculated to evaluate method robustness.

To evaluate stability, the menotropins for injection solution was placed in a sample compartment at 10 °C and injected for analysis at 0, 6, 12, 24, 36, and 48 h. The RSD of the α- and β-subunit peak area were calculated to assess sample stability.

To evaluate limit of detection and limit of quantification, the rhFSH stock solution was diluted, and the LOD and LOQ were determined at signal-to-noise ratios of 3:1 and 10:1, respectively.

#### 2.3.3 Chromatographic conditions

A Vydac 214 TP C4 column (4.6 mm × 250 mm, 5 μm) was used to establish the chromatographic conditions. Mobile phase A consisted of 0.2 mol/L phosphate buffer (pH 2.5), prepared by dissolving 27.2 g of potassium dihydrogen phosphate in 900 mL of water, adjusting the pH to 2.5 with 85% phosphoric acid, and diluting to 1,000 mL with water. Mobile phase B was an 80% acetonitrile solution. The elution gradient was programmed as follows: 0–10 min, 18%→26% B; 10–35 min, 26%→40% B; 35–36 min, 40%→80% B; 36–56 min, 80% B; and 56–56.1 min, 80%→18% B. The detection wavelength was set at 210 nm, the flow rate was 1.0 mL/min, the column temperature was maintained at 30 °C, and the injection volume was 200 μL.

### 2.4 Analysis of menotropins components

#### 2.4.1 Intact molecular weight analysis

The collected peak fractions were centrifuged and concentrated, then exchanged into approximately 150 μL of a 25 mM ammonium bicarbonate solution using a 3-kDa molecular weight cut-off (MWCO) ultrafiltration device. A 50-μL aliquot of the resulting solution was treated with 1 μL of each of the reagents in a deglycosylation kit and incubated at 37 °C for 16 h. Finally, 1 μL of 1 M DTT solution was added, and the mixture was reduced at 37 °C for 1 h. The prepared sample was then subjected to mass spectrometric analysis.

#### 2.4.2 Enzymatic digestion for peptide analysis

Five vials of menotropins for injection were dissolved in 400 μL of water. The solution was exchanged into a 25 mM ammonium bicarbonate solution using a 3-kDa MWCO ultrafiltration device (centrifuged at 15,000 rpm for 10 min, the exchange and centrifugation were both repeated three times) and concentrated to a final concentration of 1 mg/mL. A 100-μL aliquot of this solution was mixed with 100 μL of 1% (w/v) Rapigest SF surfactant and incubated at 57 °C for 15 min. Then, 2 μL of 1 M DTT was added, and the mixture was incubated at 57 °C for 30 min before cooling to room temperature. Subsequently, 4 μL of 1 M iodoacetamide solution was added, and the solution was reacted at room temperature for 30 min in the dark. Finally, 4 μL of 1 mg/mL trypsin solution was added, and the mixture was incubated overnight at 37 °C to complete digestion. The resulting peptide fragments were analyzed by LC-MS/MS, and the data were searched against the UniProt database.

#### 2.4.3 LC-MS/MS analytical method

An ACQUITY UPLC® Peptide CSH C18 column (2.1 mm × 150 mm, 1.7 μm) was used in this analysis. The mobile phase consisted of 0.1% formic acid in water (A) and 0.1% formic acid in acetonitrile (B), with the following gradient program: 0–3 min, 1% B; 3–10 min, 1%→7% B; 10–80 min, 7%→32% B; 80–85 min, 32%→98% B; 85–90 min, 98% B; 90–90.1 min, 98%→1% B; and 90.1–100 min, 1% B. The flow rate was 0.2 mL/min, the column temperature was maintained at 60 °C, and the detection wavelength was 214 nm.

Electrospray ionization was operated in positive ion mode with MSe acquisition. The scan range was m/z 50–2000. The key parameters were as follows: collision energy 80 V; 40 V cone voltage; 3 kV capillary voltage; 120 °C capillary temperature; 350 °C desolvation temperature; 50 L/h cone gas flow rate; 800 L/h desolvation gas flow rate; and 50 μL injection volume.

### 2.5 FSH biological potency

#### 2.5.1 Animals and ethics

The design of the experiment was approved by the ethics committee of the National Institute for Food and Drug Control (NIFDC).Sprague-Dawley (SD) rats, Specific Pathogen Free (SPF)-grade, female, aged 21–23 days with body weights of 45–60 g, were obtained from Animal Center of the National Institutes for Food and Drug Control (NIFDC). For a single experiment, a total of 48 animals were used. Rats were housed in the NIFDC Laboratory Animal Facility under the following conditions: barrier environment with sterile air via secondary filtration; 12-h light/dark cycle (automatically controlled illumination); constant temperature (23 °C–25 °C) and humidity (50%–55%); maintenance in individually ventilated cages (IVC). Female rats were randomized into a control group and test groups; each was further divided into three dose levels (high, medium, low).

#### 2.5.2 Reference standard and test solution preparation

The bioassay for FSH was performed in accordance with General Chapter 1216 of the Chinese Pharmacopoeia (ChP, Vol. IV, Chinese Pharmacopoeia Commission, 2020a). An appropriate amount of BSA was dissolved in physiological saline to prepare a 1 mg/mL solution, followed by pH adjustment to 7.2 ± 0.2 using 1 mol/L NaOH. This solution was then utilized to dissolve the rhCG reference standard, yielding a vehicle containing 20 IU/mL of hCG.

Both the menotropins standard and test samples were diluted with the vehicle to establish three dose groups: high (3.0 IU/mL, SH), medium (1.5 IU/mL, SM), and low (0.75 IU/mL, SL). Rats in each group received daily subcutaneous injections of 0.5 mL at the cervical region for three consecutive days. Twenty-four hours after the final administration,the rats were transferred to a euthanasia chamber and exposed to a flow of 100% CO_2_ at a rate displacing 30% of the chamber volume per minute. Death was confirmed by the cessation of breathing, followed by cervical dislocation as a secondary measure to ensure euthanasia. Subsequent necropsy and experimental procedures were then performed.Body weights were recorded, and bilateral ovaries were surgically excised. After carefully removing adherent tissues and blotting with filter paper to absorb residual fluid, the ovaries were weighed to a precision of 0.1 mg.

#### 2.5.3 Statistical analysis

Using ovarian weight (mg per 10 g body weight) as the response value, the potency and experimental error were calculated according to the parallel-line three-by-three assay design for quantitative responses specified in General Rule 1431 of the Chinese Pharmacopoeia (2025 Edition, Volume IV). Validity test requirements were met, including significant regression (P < 0.01), no significant deviation from parallelism (P > 0.05), no significant quadratic curvature (P > 0.05), and no significant reverse quadratic curvature (P > 0.05). Furthermore, and the mean fiducial limit percentage (FL%) was controlled below 45%.

### 2.6 LH biological potency

#### 2.6.1 Animals and ethics

The design of the experiment was approved by the ethics committee of the National Institute for Food and Drug Control (NIFDC). Sprague-Dawley (SD) rats, Specific Pathogen Free (SPF)-grade, male, aged 21–23 days with body weights of 40–55 g, were obtained from Animal Center of the National Institutes for Food and Drug Control (NIFDC).For a single experiment, a total of 48 animals were used. Rats were housed in the NIFDC Laboratory Animal Facility under the following conditions: barrier environment with sterile air via secondary filtration; 12-h light/dark cycle (automatically controlled illumination); constant temperature (23 °C–25 °C) and humidity (50%–55%); maintenance in individually ventilated cages (IVC). Male rats were randomized into a control group and test groups; each was further divided into three dose levels (high, medium, low).

#### 2.6.2 Reference standard and test solution preparation

The bioassay for LH was performed in accordance with General Chapter 1217 of the Chinese Pharmacopoeia (ChP, Vol. IV, Chinese Pharmacopoeia Commission, 2020b). An appropriate amount of BSA was dissolved in physiological saline to prepare a 1 mg/mL solution, followed by pH adjustment to 7.2 ± 0.2 using 1 mol/L NaOH. Both the menotropins standard and test samples were diluted with the vehicle to establish three dose groups: high (15 IU/mL, SH), medium (7.5 IU/mL, SM), and low (3.75 IU/mL, SL). Rats in each group received daily subcutaneous injections of 0.5 mL at the cervical region for four consecutive days. Twenty-four hours after the final administration,the rats were transferred to a euthanasia chamber and exposed to a flow of 100% CO_2_ at a rate displacing 30% of the chamber volume per minute. Death was confirmed by the cessation of breathing, followed by cervical dislocation as a secondary measure to ensure euthanasia. Subsequent necropsy and experimental procedures were then performed. Body weights were recorded, and seminal vesicles were surgically excised. After carefully removing adherent tissues and blotting with filter paper to absorb residual fluid, the seminal vesicles were weighed to a precision of 0.1 mg.

#### 2.6.3 Statistical analysis

Using seminal vesicles weight (mg per 10 g body weight) as the response value, the potency and experimental error were calculated according to the parallel-line three-by-three assay design for quantitative responses specified in General Rule 1431 of the Chinese Pharmacopoeia (2025 Edition, Volume IV). Validity test requirements were met, including significant regression (P < 0.01), no significant deviation from parallelism (P > 0.05), no significant quadratic curvature (P > 0.05), and no significant reverse quadratic curvature (P > 0.05). Furthermore, the mean fiducial limit percentage (FL%) was controlled below 35%.

## 3 Results

### 3.1 Establishment of the chromatographic method

FSH, hCG, and LH are heterodimers formed by the non-covalent binding of a common α-subunit and distinct β-subunits; here, we aimed to develop a chromatographic method to effectively separate the α-subunit, β-subunit, oxidized α-subunit, and oxidized β-subunit peaks of these three active components. This enabled a quantitative determination of the protein content of each active ingredient using an external standard method.

**FIGURE 1:**
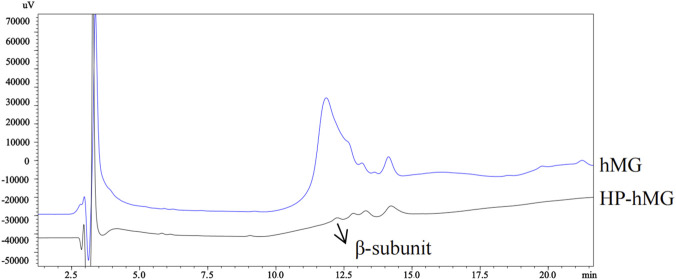
Chromatograms of menotropins and HP-hMG obtained using an imported drug registration standard method.

The oxidized subunit assay method from the imported drug registration standard for HP-hMG for injection (JX20220062) was used to analyze both the conventional menotropins and highly purified menotropin. As shown in Figure 1, the β-subunit of conventional menotropins could not be adequately resolved from adjacent peaks. In contrast to conventional menotropins, HP-hMG has higher purity, whereas conventional menotropins has lower purity and contains numerous protein impurities (Zwart-van Rijkom, 2002). Thus, this method was not suitable for separating the subunits of conventional menotropins.

**FIGURE 2:**
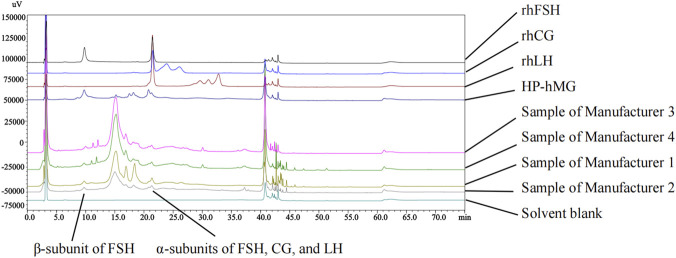
Chromatograms of various test solutions according to an established method described in the study.

**FIGURE 3:**
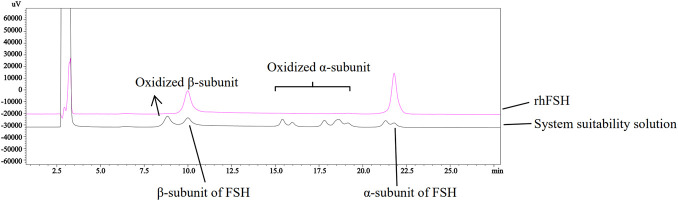
Chromatogram of the system suitability solution and rhFSH (α-subunit of FSH: represents the α-subunit of FSH; β-subunit of FSH: represents the specific β-subunit of FSH; Oxidized α-subunit: represents the oxidized form of the α-subunit; Oxidized β-subunit: represents the oxidized form of the β-subunit).

Therefore, the chromatographic conditions of a method were established to achieve this separation. As illustrated in Figures 2, 3, the β-subunit of FSH, the oxidized α-subunit, and the α-subunit of FSH, LH and hCG were effectively separated. Moreover, no interference from LH or hCG was observed at the retention time of the FSH β-subunit peak.

### 3.2 Identification of menotropins components

The chromatogram of the menotropins solution revealed a significant unknown peak between the β-subunit and α-subunit peaks. It was speculated that, in addition to the oxidized α-subunit, this peak contained unresolved unknown protein impurities, producing a complex composition that presented challenges for quality control studies of this product. To ensure the accurate identification of each peak component and enable precise quantification of principal components content, each peak fraction was collected, and the molecular weights were analyzed to determine the identity of the components.

**FIGURE 4:**
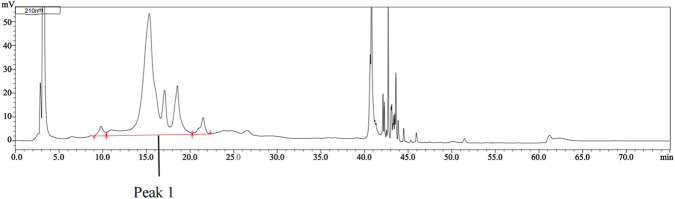
Chromatogram of the menotropins solution determined by RP-HPLC. The figure illustrates the separation of the FSH α-subunit and β-subunit from a large number of protein impurities (as shown in the Peak 1 region) under complex matrix interference. Details of the solution preparation and chromatographic conditions can be found in Sections 2.3.1 and 2.3.3, respectively.

**FIGURE 5:**
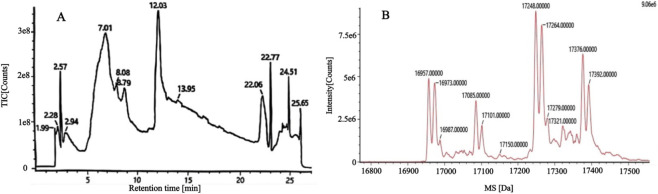
Total ion current **(A)** and first-order mass spectrum **(B)** of peak 1 components.

Peak 1 from the chromatogram of the menotropins solution (Figure 4) was collected, desalted, and subjected to deglycosylation. Figure 5 depicts the complex composition of peak 1, containing multiple distinct proteins. Menotropins was then digested with trypsin, and the peptide information acquired via LC-MS/MS was searched against the UniProt database to obtain protein composition data. The detailed data are listed in Supplementary Table 1. The search results indicated that menotropins contained active ingredients that included FSH, LH, and hCG, and dozens of protein impurities with molecular weights ranging from 13 kDa to 130 kDa. The variety of protein impurities was extensive, and their content was relatively high. This created interference and posed significant challenges in the quality control and analysis of the active components. The present method effectively separated the FSH β-subunit from the protein impurities and was used to quantify the FSH protein content.

### 3.3 Methodological validation

The validation items, in accordance with ICH Q2 (R1) Analytical Procedure Validation: Text and Methodology and General Chapter 9101″Guidelines for Validation of Analytical Procedures” in Volume IV of the Pharmacopoeia of the People’s Republic of China (2025 Edition), include Specificity, Precision, Linearity and Range, Accuracy, Robustness, Stability, Limit of Detection (LOD), and Limit of Quantification (LOQ).

#### 3.3.1 Specificity

**FIGURE 6:**
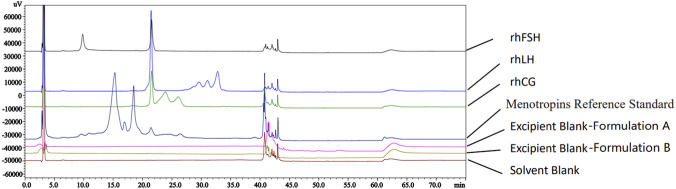
Chromatogram of the specificity evaluation solutions.

As shown in Figure 6, neither the solvent blank nor the excipients interfered with the detection of the FSH β-subunit and α-subunit in menotropins. The β-subunits of hCG and LH did not exhibit elution peaks at the same retention position, confirming that the method demonstrated satisfactory specificity.

#### 3.3.2 Precision

To evaluate precision, five consecutive injections of the linearity sample solution at a concentration of 0.01 mg/mL yielded an RSD of 0.25% for the α-subunit peak area, an RSD of 1.6% for the peak area of the β-subunit, indicating good precision.

#### 3.3.3 Linearity range

A linear regression was performed with the mass concentration of the α-subunit as the abscissa and the peak area as the ordinate. The linear regression equation of the α-subunit was y = 314515834x−110417, with a coefficient of determination (*R* ^2^) of 0.9983, demonstrating good linearity.

A linear regression analysis was performed with the mass concentration of the β-subunit as the abscissa and the peak area as the ordinate. The linear regression equation for the β-subunit was y = 196427140x −59603, with a coefficient of determination (*R* ^2^) of 0.9981, demonstrating good linearity. The linear relationship plot is shown in Supplementary Figure 1.

#### 3.3.4 Accuracy

As shown in Table 1, the recovery rates of total α-subunits ranged from 90.0% to 110.0%, with an RSD of less than 2.0%, demonstrating that the method provided satisfactory accuracy for the quantification of the total α-subunit.

**TABLE 1:** Recovery rate determination of the α-subunit**.**

Solution concentration level	Theoretical value (μg/mL)	Measured value (μg/mL)	Recovery rate (%)	Average recovery rate (n = 3) (%)	Average recovery rate (n = 9) (%)
70% spiked solution	3.171	3.126	98.6	97.4 (RSD = 1.35%)	97.9 (RSD = 0.80%)
	3.171	3.096	97.6		
	3.171	3.045	96.0		
100% spiked solution	4.530	4.428	97.8	97.9 (RSD = 0.12%)	
	4.530	4.440	98.0		
	4.530	4.441	98.0		
130% spiked solution	5.889	5.758	98.1	98.2 (RSD = 0.52%)	
	5.889	5.791	97.8		
	5.889	5.816	98.8		

As summarized in Table 2, the recovery rates of the FSH β-subunit ranged from 90.0% to 110.0%, with an RSD of less than 2.0%, confirming that the method provided satisfactory accuracy for FSH β-subunit quantification.

**TABLE 2:** Recovery rate determination of the FSH β-subunit.

Solution concentration level	Theoretical value (μg/mL)	Measured value (μg/mL)	Recovery rate (%)	Average recovery rate (n = 3) (%)	Average recovery rate (n = 9) (%)
70% spiked solution	3.829	3.980	103.9	102.8 (RSD = 0.98%)	102.0 (RSD = 1.50%)
	3.829	3.901	101.9		
	3.829	3.931	102.7		
100% spiked solution	5.470	5.557	101.6	102.4 (RSD = 1.02%)	
	5.470	5.584	102.1		
	5.470	5.668	103.6		
130% spiked solution	7.111	7.130	100.3	100.6 (RSD = 1.71%)	
	7.111	7.045	99.1		
	7.111	7.285	102.5		

#### 3.3.5 Robustness

The menotropins for injection solution was analyzed using three different chromatographic columns. The RSD of the α-subunit peak area was 5.3%, FSH β-subunit peak area was 1.1%, indicating good method robustness.

#### 3.3.6 Stability

To analyze stability, a sample was stored at 10 °C and then analyzed at 0, 6, 12, 24, 36, and 48 h. The RSD of the α-subunit peak area was 1.1%, β-subunit peak area was 1.9%, demonstrating that the sample solution remained stable for up to 48 h when stored at these conditions.

#### 3.3.7 Limit of detection and limit of quantification

The LOD and LOQ were determined at signal-to-noise ratios of 3:1 and 10:1, respectively. The LOD and LOQ of the α-subunit and FSH β-subunit were found to be 3 ng and 9 ng, respectively.

### 3.4 Determination of total α-subunits and FSH content in menotropins and its preparations

The developed method was applied to the analysis of 4 batches of menotropins raw materials and 69 batches of commercial menotropins for injection collected from various manufacturers. The total α-subunits content in menotropins raw materials was determined to range from 0.9% to 1.4%. The FSH content in menotropins raw materials ranged from 3.0% to 4.1%. This result was consistent with the component analysis, indicating that FSH, LH and hCG constitute trace yet high specific activity component in menotropins raw materials, whereas predominant protein impurities account for the majority of protein content.

For the finished preparations, the total α-subunits content per vial was measured as follows: manufacturer 1 (29 batches), 1.43–1.92 μg/vial; manufacturer 2 (16 batches), 1.30–1.45 μg/vial; manufacturer 3 (9 batches), 1.33–1.51 μg/vial; and manufacturer 4 (15 batches), and 1.39–2.11 μg/vial. The RSD were 7.1%, 3.4%, 4.4%, and 12.5% for manufacturers 1, 2, 3, and 4, respectively. The FSH content per vial was determined as follows: manufacturer 1 (29 batches), 4.38–5.13 μg/vial; manufacturer 2 (16 batches), 4.43–4.99 μg/vial; manufacturer 3 (9 batches), 4.35–5.44 μg/vial; and manufacturer 4 (15 batches), 3.80–5.36 μg/vial. The relative standard deviations of the FSH content across batches were 3.5%, 3.5%, 6.3%, and 12.6% for manufacturers 1, 2, 3, and 4, respectively.

**FIGURE 7:**
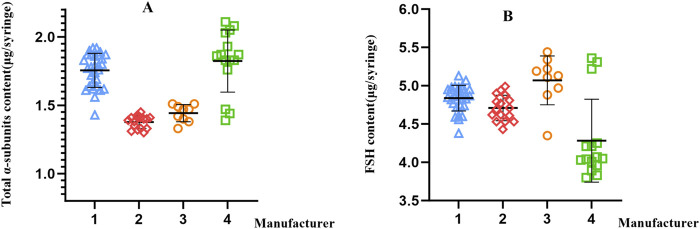
Scatter plots of total α-subunits content **(A)** and FSH content **(B)** using the developed method of samples from different manufacturers.

The scatter plot of these results is shown in Figures 7A,B. The tota α-subunits content of the products from manufacturer 2 and manufacturer 3 were relatively clustered, indicating good batch-to-batch consistency. The FSH content of the products from manufacturer 1 and manufacturer 2 were relatively clustered, indicating good batch-to-batch consistency. Manufacturer 4 showed a wider range of both tota α-subunits and FSH content.

### 3.5 Determination of LH and FSH biological potency in menotropins and its preparations

The FSH and LH biological potency of all 69 batches from the four manufacturers met the specified requirements. The FSH biological potency per vial was determined as follows: manufacturer 1 (29 batches), 79%–124%; manufacturer 2 (16 batches), 80%–125%; manufacturer 3 (9 batches), 80%–115%; and manufacturer 4 (15 batches), 77%–119%. The relative standard deviations of the FSH potency across batches were 12.0%, 13.1%, 11.9%, and 15.9% for manufacturers 1, 2, 3, and 4, respectively. The LH biological potency per vial was determined as follows: manufacturer 1 (29 batches),80%–127%; manufacturer 2 (16 batches), 79%–111%; manufacturer 3 (9 batches), 81%–112%; and manufacturer 4 (15 batches), 81%–116%. The relative standard deviations of the FSH potency across batches were 12.6%, 9.9%, 11.9%, and 10.6% for manufacturers 1, 2, 3, and 4, respectively.

**FIGURE 8:**
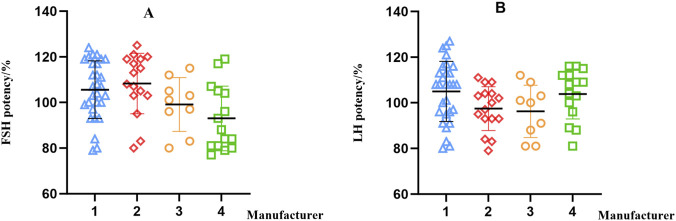
Scatter plots of FSH **(A)** and LH **(B)** biological potency determined by *in vivo* bioactivity assays of samples from different manufacturers.

The scatter plot of these results is shown in Figures 8A,B. The FSH and LH *in vivo* bioactivity assay exhibited high variability, leading to more dispersed results. Among the 69 batches of menotropins for injection, several batches were samples obtained from different distribution stages with the same batch number. For these samples, the total α-subunits content and FSH protein content per vial were basically consistent, while significant deviations were observed in FSH and LH potency. These results demonstrate that the *in vivo* animal model method possesses high variability and fails to reflect the true batch-to-batch variation, whereas the HPLC method developed in this study shows excellent reproducibility.

### 3.6 Purity determination of menotropins and its preparations

The established HPLC method was further applied to purity determination using an area normalization approach, where the percentage of combined α- and β-subunit peak areas represented the purity value. For finished preparations, purity results were as follows manufacturer 1 (29 batches), 6.9%–8.6%; manufacturer 2 (16 batches), 9.4%–10.9%; manufacturer 3 (9 batches), 5.6%–6.3%; and manufacturer 4 (15 batches), and 5.8%–42.4%. The RSD were 5.7%, 4.3%, 3.8%, and 46.6% for manufacturers 1, 2, 3, and 4, respectively.

**FIGURE 9:**
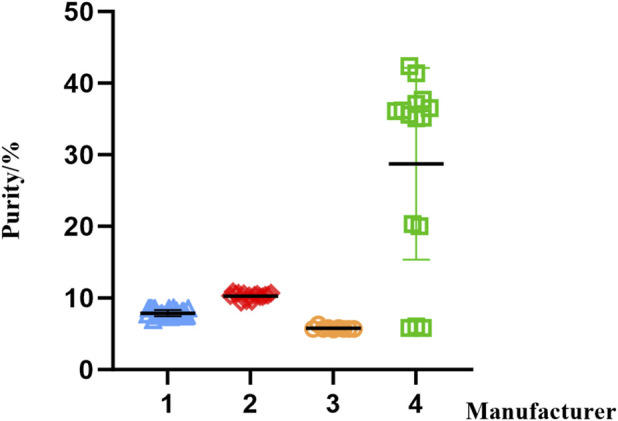
Purity of samples from different manufacturers.

**FIGURE 10:**
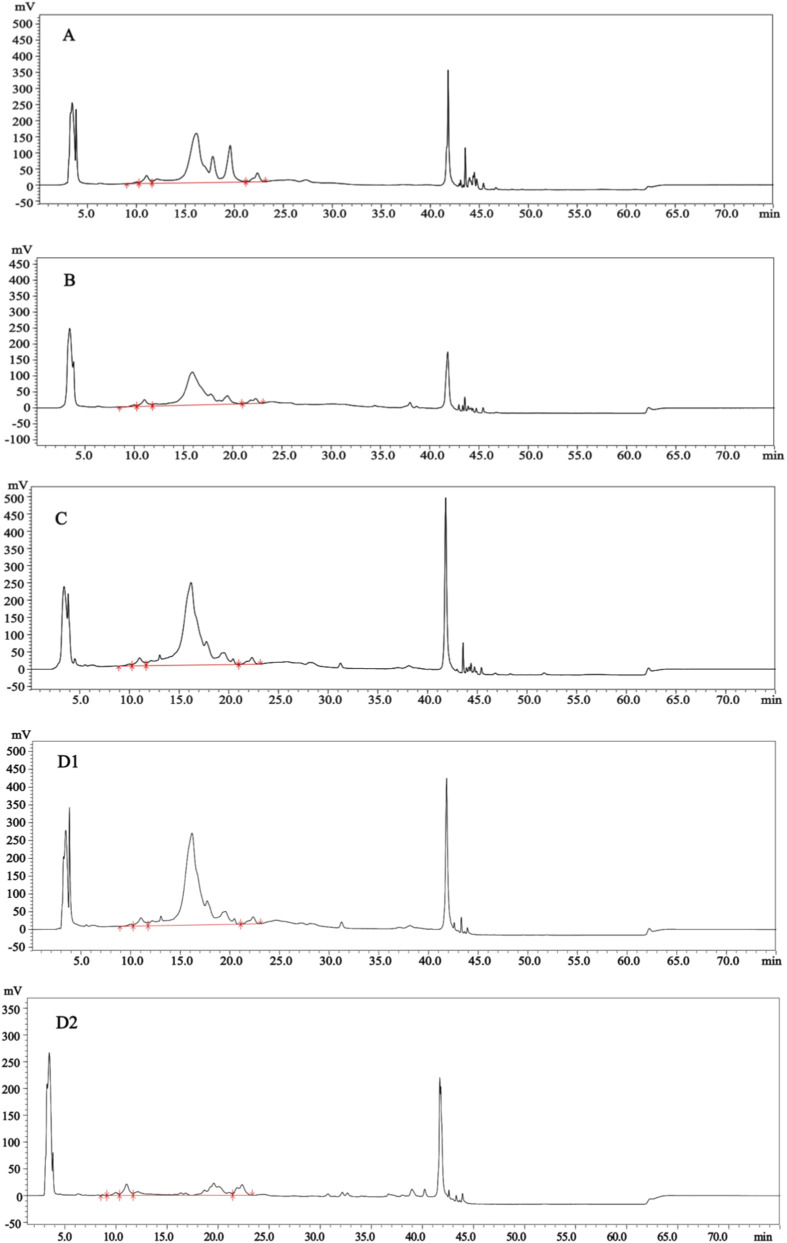
Representative chromatograms for purity assessment of four manufacturers **(A)** represents Manufacturer 1 samples; **(B)** represents Manufacturer 2 samples; **(C)** represents Manufacturer 3 samples; **(D1,D2)** represent different batches of samples from Manufacturer 4).

The scatter plot of these results is shown in Figure 9. Manufacturers 1–3 demonstrated good inter-batch consistency in purity, whereas Manufacturer 4 exhibited substantial batch-to-batch variability. As shown in Figure 10 (representative chromatograms), significant impurity peaks were observed in samples from Manufacturers 1–3 and some batches of Manufacturer 4 (Figure 10D1). In contrast, another typical chromatogram from Manufacturer 4 (Figure 10D2) showed minimal impurity peaks and higher purity. This method successfully discriminates purity variations in menotropins, proving applicable for quality control of product purity.

## 4 Discussion

Menotropins is a complex mixture of extracted gonadotropins (FSH, pituitary and urinary hCG, and LH), containing up to 20% non-gonadotropic proteins and approximately 40% oxidized forms of FSH, resulting in low purity (Bassett and Driebergen, 2005; Lispi et al., 2006; Bassett et al., 2009; Lunenfeld et al., 2019). This complexity poses significant challenges for the precise quantification of its active components. The primary adverse reaction associated with menotropins is ovarian hyperstimulation syndrome, characterized by ovarian enlargement, abdominal distension, abdominal pain, nausea, as well as multiple pregnancies and preterm labor (Haddad, 1999). Due to the high specific activity of its active components, even minor fluctuations in the content of menotropins can significantly impact potency. The current pharmacopeial standards rely solely on *in vivo* bioassays for potency control, which exhibit high variability. Therefore, establishing an accurate quantitative method for the active components is crucial for guiding clinical dosing and reducing adverse reactions.While the analysis of HP-hMG or recombinant gonadotropins has been reported (Capolupo et al., 2024; Lispi et al., 2006), no literature to date has described the use of RP-HPLC for the quantification of active components in conventional menotropins.Experimental results demonstrate that existing HP-hMG registration standard methods using RP-HPLC cannot effectively separate the β-subunit from adjacent impurity peaks in menotropins. The complexity of conventional menotropins far exceeds that of HP-hMG and recombinant products, as they contain a very low proportion of active ingredients and a large number of protein impurities between 13 kDa and 130 kDa. In this study, we overcame the challenges posed by the complex composition, trace levels, and structural complexity of active components in menotropins, achieving effective separation of the α-subunit, which is common to three trace, high-specific-activity components, and the specific β-subunit from both protein impurities and the oxidized α-subunit, thereby enabling accurate quantification.

The HPLC method established in this study enables the simultaneous quantification of the total α-subunits content, FSH content, and purity. The FSH content was quantified using the β-subunit as the primary peak, which assumes the absence of subunit oxidation. However, menotropins contains a significant amount of oxidized α-subunits, and such oxidized glycoproteins exhibit reduced bioactivity (Nevelli et al., 2023). As a result, controlling solely for the FSH content does not accurately regulate the therapeutically effective components. The total α-subunits content was quantified using the shared α-subunit peak (i.e., the peak common to the FSH, LH, and hCG active components) that resolves oxidized α-subunits. The simultaneous assessment of the total α-subunits content, FSH content, and purity provides a more rational evaluation of active component levels for enhancing product quality control.

A total of 69 batches of domestic menotropins for injection, spanning three key circulation stages (manufacturers, distributors, and end-users), were collected to comprehensively reflect real-world product quality profiles. This extensive analysis of commercial batches from diverse sources underscores the practical utility of our approach for the rigorous quality surveillance of menotropins.Using an in-house validated HPLC method, we quantified the total α-subunits and FSH protein content and purity in these batches, with results systematically compared against FSH and LH potency data generated by the standard *in vivo* bioassay. This head-to-head comparison revealed that the HPLC method, with its superior precision, specificity, and reproducibility, not only enables accurate quantification of total α-subunits and FSH in complex menotropins matrices but also provides a direct, objective measure of active ingredient content—addressing the limitations of the bioassay, such as high variability and poor batch-to-batch discriminative power. Importantly, the method’s robustness, as demonstrated by consistent performance across 69 diverse samples, confirms its suitability for routine quality control of menotropins formulations. By offering a reliable means to monitor α-subunits and FSH content and purity throughout the product lifecycle, this HPLC method holds significant practical value for ensuring batch consistency, optimizing manufacturing processes, and ultimately enhancing the safety and efficacy of menotropins-based therapies.Consequently, this method provides a highly discriminative quality control solution for menotropins, which are complex extracted protein drugs. It can be effectively utilized for inter-batch consistency control, raw material selection, and stability monitoring. Furthermore, it enables rapid, accurate, and sensitive quality control and has undergone large-scale applicability validation.

In conclusion, given the intricacies of the menotropins composition, the extremely low content of active ingredients, and substantial interference, a robust HPLC method using the FSH α- and β-subunit peak as the primary marker was developed, based on an external standard approach. The method demonstrated excellent separation, high specificity, and good reproducibility, enabling accurate quantification of FSH protein content. With standardized validation, this method is suitable for enhancing quality standards of menotropins products, thereby improving the quality control of this complex preparation and ensuring medication safety.

## Data Availability

The raw data supporting the conclusions of this article will be made available by the authors, without undue reservation.

## References

[B1] Bassett R. Driebergen R. (2005). Continued improvements in the quality and consistency of follitropin alfa, recombinant human FSH. Reprod. Biomed. Online 10, 169–177. 10.1016/s1472-6483(10)60937-6 15823219

[B2] Bassett R. Lispi M. Ceccarelli D. Grimaldi L. Mancinelli M. Martelli F. (2009). Analytical identification of additional impurities in urinary-derived gonadotrophins. Reprod. Biomed. Online 19, 300–313. 10.1016/s1472-6483(10)60163-0 19778474

[B3] Bergandi L. Canosa S. Carosso A. R. Paschero C. Gennarelli G. Silvagno F. (2020). Human recombinant FSH and its biosimilars: clinical efficacy, safety, and cost-effectiveness in controlled ovarian stimulation for *in vitro* fertilization. Pharmaceuticals 13, 136. 10.3390/ph13070136 32605133 PMC7407829

[B4] Butt W. R. Crooke A. C. Cunningham F. J. Evans A. J. (1961). Chemical reactions which affect the biological activity of human gonadotrophins. Biochem. J. 79, 64–70. 10.1042/bj0790064 13689464 PMC1205548

[B5] Cahoreau C. Klett D. Combarnous Y. (2015). Structure–function relationships of glycoprotein hormones and their subunits’ ancestors. Front. Endocrinol. 6, 26. 10.3389/fendo.2015.00026 25767463 PMC4341566

[B6] Capolupo A. Petrocchi S. Melchiorre M. Jonas K. D’Hooghe T. Hanyaloglu A. (2024). Analytical investigation of the profile of human chorionic gonadotropin in highly purified human menopausal gonadotrophin preparations. Int. J. Mol. Sci. 25, 1801. 10.3390/ijms25031801 39273352 PMC11395176

[B7] Chen L.-H. Chin T.-H. Huang S.-Y. Yu H.-T. Chang C.-L. Huang H.-Y. (2021). Supplementation with human menopausal gonadotropin in the gonadotropin-releasing hormone antagonist cycles of women with high AMH: pregnancy outcomes and serial hormone levels. Taiwan. J. Obstet. Gynecol. 60, 739–744. 10.1016/j.tjog.2021.05.027 34247817

[B8] Chinese Pharmacopoeia Commission (2020a). General chapter 1216: biological assay of follicle stimulating hormone, in pharmacopoeia of the people’s Republic of China, IV. Beijing: China Medical Science Press, 227–228.

[B9] Chinese Pharmacopoeia Commission (2020b). General chapter 1217: biological assay of luteinising hormone, in pharmacopoeia of the people’s Republic of China, IV. Beijing: China Medical Science Press, 228.

[B10] Chinese Pharmacopoeia Commission (2025). “Human follitropin for injection,” in Pharmacopoeia of the people’s Republic of China, 402–409.

[B11] Driebergen R. Baer G. (2003). Quantification of follicle stimulating hormone (follitropin alfa): is *in vivo* bioassay still relevant in the recombinant age? Curr. Med. Res. Opin. 19, 41–46. 10.1185/030079902125001344 12661779

[B12] Haddad R. (1999). Physical and psychological changes associated with controlled ovarian hyperstimulation in women undergoing assisted reproductive techniques. Byblos, Lebanon: Lebanese American University. 10.26756/th.1999.14

[B13] Khair A. Brown T. Markert M. Barsøe C. R. Daftary G. S. Heiser P. W. (2023). Highly purified human menopausal gonadotropin (HP-hMG) *versus* recombinant follicle-stimulating hormone (rFSH) for controlled ovarian stimulation in US predicted high-responder patients: a cost-comparison analysis. PharmacoEconomics - Open 7, 851–860. 10.1007/s41669-023-00429-8 37480456 PMC10471533

[B14] Leão R. de B. F. Esteves S. C. (2014). Gonadotropin therapy in assisted reproduction: an evolutionary perspective from biologics to biotech. Clin. Sao Paulo Braz 69, 279–293. 10.6061/clinics/2014(04)10 24714837 PMC3971356

[B15] Lispi M. Bassett R. Crisci C. Mancinelli M. Martelli F. Ceccarelli D. (2006). Comparative assessment of the consistency and quality of a highly purified FSH extracted from human urine (urofollitropin) and a recombinant human FSH (follitropin α). Reprod. Biomed. Online 13, 179–193. 10.1016/s1472-6483(10)60613-x 16895630

[B16] Lispi M. Humaidan P. Bousfield G. R. D’Hooghe T. Ulloa-Aguirre A. (2023). Follicle-stimulating hormone biological products: does potency predict clinical efficacy? Int. J. Mol. Sci. 24, 9020. 10.3390/ijms24109020 37240364 PMC10218858

[B17] Lunenfeld B. (2012). Gonadotropin stimulation: past, present and future. Reprod. Med. Biol. 11, 11–25. 10.1007/s12522-011-0097-2 29699102 PMC5906949

[B18] Lunenfeld B. Bilger W. Longobardi S. Alam V. D’Hooghe T. Sunkara S. K. (2019). The development of gonadotropins for clinical use in the treatment of infertility. Front. Endocrinol. 10, 429. 10.3389/fendo.2019.00429 31333582 PMC6616070

[B19] Muqaku L. Dorrer V. Noe M. Noe C. R. Nebija D. (2024). Quality evaluation of highly purified human menopausal gonadotropin preparations by means of gel electrophoresis and mass spectrometry. Pharm 79, 57–63. 10.1691/ph.2024.4003 38872273

[B20] Nevelli F. Palmese A. Gleixner R. Peroglio F. D’Acunto C.-W. Dadone A. (2023). Biological assay to determine gonadotropin potency: from *in vivo* to *in vitro* sustainable method. Int. J. Mol. Sci. 24, 8040. 10.3390/ijms24098040 37175746 PMC10178553

[B21] Pacchiarotti A. Selman H. Valeri C. Napoletano S. Sbracia M. Antonini G. (2016). Ovarian stimulation protocol in IVF: an up-to-date review of the literature. Curr. Pharm. Biotechnol. 17, 303–315. 10.2174/1389201017666160118103147 26775651

[B22] Practice Committee of American Society for Reproductive Medicine, Birmingham, Alabama (2008). Gonadotropin preparations: past, present, and future perspectives. Fertil. Steril. 90, S13–S20. 10.1016/j.fertnstert.2008.08.031 19007609

[B23] Revelli A. Pettinau G. Basso G. Carosso A. Ferrero A. Dallan C. (2015). Controlled ovarian stimulation with recombinant-FSH plus recombinant-LH vs. human menopausal gonadotropin based on the number of retrieved oocytes: results from a routine clinical practice in a real-life population. Reprod. Biol. Endocrinol. 13, 77. 10.1186/s12958-015-0080-6 26209525 PMC4514947

[B24] Ulloa-Aguirre A. Zariñán T. Jardón-Valadez E. Gutiérrez-Sagal R. Dias J. A. (2018). Structure-function relationships of the follicle-stimulating hormone receptor. Front. Endocrinol. 9, 707. 10.3389/fendo.2018.00707 30555414 PMC6281744

[B25] Wolfenson C. Groisman J. Couto A. S. Hedenfalk M. Cortvrindt R. G. Smitz J. E. (2005). Batch-to-batch consistency of human-derived gonadotrophin preparations compared with recombinant preparations. Reprod. Biomed. Online 10, 442–454. 10.1016/s1472-6483(10)60819-x 15901450

[B26] Yetkinel S. Aytaç P. Ç. Durdağ G. D. Yağınç D. A. Kılıçdağ E. B. Şimşek E. (2024). Comparison of highly purified human menopausal gonadotropin and recombinant follicle stimulating hormone use in patients undergoing *in vitro* fertilization with progestin-primed ovarian stimulation protocol: a single center retrospective analysis. Arch. Gynecol. Obstet. 310, 2657–2662. 10.1007/s00404-024-07756-z 39358454

[B27] Zwart-van Rijkom J. E. F. Broekmans F. J. Leufkens H. G. M. (2002). From HMG through purified urinary FSH preparations to recombinant FSH: a substitution study. Hum. Reprod. 17, 857–865. 10.1093/humrep/17.4.857 11925373

