# Supplementary figures and images for: Quantitative determination of trace principal components with high specific activity in menotropins
Source: Front Bioeng Biotechnol. 2026 May 4;14:1783311. doi: 10.3389/fbioe.2026.1783311 (PMC13180910; doi:10.3389/fbioe.2026.1783311)

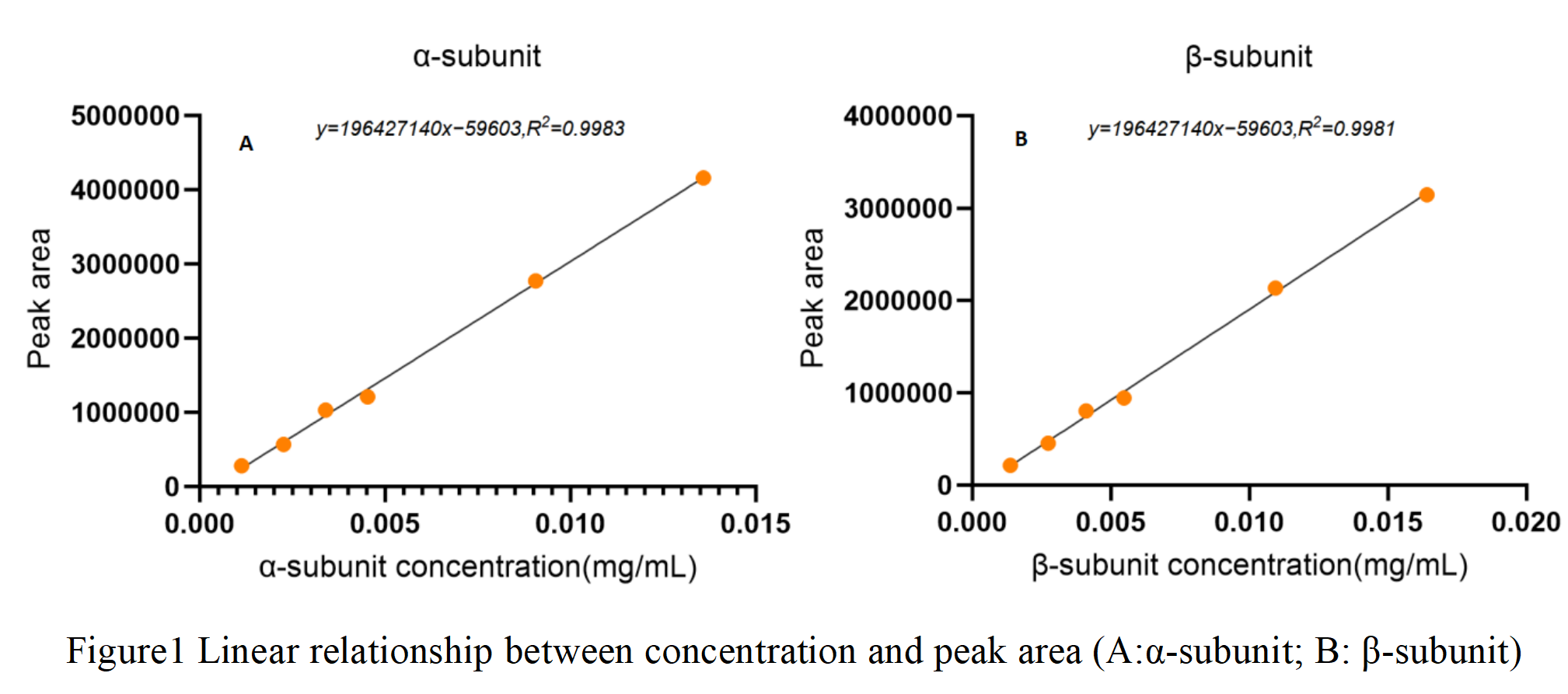

Supplement: Supplementary file 1 [file Image1.tif]
