# Supplementary material for: Quantitative determination of trace principal components with high specific activity in menotropins
Source: Front Bioeng Biotechnol. 2026 May 4;14:1783311. doi: 10.3389/fbioe.2026.1783311 (PMC13180910; doi:10.3389/fbioe.2026.1783311)
Supplement: Supplementary file 2 [file Table1.doc]

Table1 The mass spectrometry analysis results of Peak 1

| Number | Molecular Weight /Da | Description | Raw abundance |
| --- | --- | --- | --- |
| 1 | 39911.9931 | Protein AMBP | 4054213.072 |
| 2 | 34486.8721 | Zinc-alpha-2-glycoprotein | 847788.7444 |
| 3 | 38631.7736 | Urokinase plasminogen activator surface receptor | 1199480.882 |
| 4 | 71362.7109 | Albumin | 80414.43949 |
| 5 | 68562.9458 | Progranulin | 216980.5022 |
| 6 | 42426.6637 | Complement decay-accelerating factor | 658991.774 |
| 7 | 39609.831 | Beta-2-glycoprotein | 1025678.77 |
| 8 | 96711.8651 | Complement component C7 | 2753363.954 |
| 9 | 81320.1098 | Fibulin-1 | 283908.61 |
| 10 | 30731.6233 | Folate receptor alpha | 1413651.521 |
| 11 | 15612.4466 | Follitropin subunit beta OS=Homo sapiens OX=9606 GN=FSHB PE=1 SV=2 | 2084478.714 |
| 12 | 43170.9064 | Protein delta homolog 2 OS=Homo sapiens OX=9606 GN=DLK2 PE=2 SV=1 | 611044.903 |
| 13 | 21560.7271 | Apolipoprotein D | 2493606.745 |
| 14 | 22130.0202 | Peptidoglycan recognition protein 1 | 2905989.652 |
| 15 | 30156.8435 | Insulin-like growth factor-binding protein 7 | 291663.3159 |
| 16 | 30334.9183 | CD27 antigen | 7985519.562 |
| 17 | 37481.4515 | Signal-regulatory protein beta-2 | 230170.3094 |
| 18 | 21152.3401 | Choriogonadotropin subunit beta variant 1 | 2006506**.**939 |
| 19 | 35590.2563 | Junctional adhesion molecule C | 489131.5417 |
| 20 | 52205.7408 | Tumor necrosis factor receptor superfamily member 1A | 231404.8201 |
| 21 | 132332.6105 | Fibulin-2 | 77934.59185 |
| 22 | 19130.2049 | Lithostathine-1-alpha | 239175.6028 |
| 23 | 13645.4629 | Glycoprotein hormones alpha chain | 269604.9448 |
| 24 | 16029.6426 | Lutropin subunit beta | 123508.5376 |
